# MicroRNA-362-5p promotes the proliferation and inhibits apoptosis of trophoblast cells via targeting glutathione-disulfide reductase

**DOI:** 10.1080/21655979.2021.1933678

**Published:** 2021-06-09

**Authors:** Cuihua Zhang, Dan Zhao

**Affiliations:** First Department of Obstetrics, Chongqing Maternal and Child Health Hospital, Chongqing, China

**Keywords:** Gestational diabetes mellitus, miR-362-5p, GSR, proliferation, apoptosis

## Abstract

Gestational diabetes mellitus (GDM), a common complication of pregnancy, harms the health of pregnant women and fetuses. MicroRNAs (miRNAs) dysregulation in placenta is involved in GDM. Herein, we explored the roles of miR-362-5p in GDM. After high glucose (HG) treated HTR-8/SVneo cells, CCK-8 and flow cytometry were conducted to assess the capability of the proliferation and apoptosis, respectively. The data demonstrated that HG inhibited proliferation and induced apoptosis of HTR-8/SVneo cells. MiR-362-5p level was reduced in HG-treated cells and placenta tissues of GDM patients, measured by qPCR. Overexpressed miR-362-5p accelerated the proliferation and restrained apoptosis of HG-treated cells. Furthermore, glutathione-disulfide reductase (GSR) was verified as a target of miR-362-5p, through TargetScan database and dual-luciferase reporter assay. GSR was upregulated in GDM placenta tissues and was negatively regulated by miR-362-5p. Enforced GSR level abolished the effects of miR-362-5p overexpression on the proliferation and apoptosis of HTR-8/SVneo cells. Furthermore, miR-362-5p increased p-PI3K, p-AKT and bcl-2, while reduced bax and cleaved caspase3, which were abolished by GSR. In conclusion, miR-362-5p promoted cell proliferation and inhibited apoptosis via targeting GSR and activating PI3K/AKT pathway. The findings mentioned above suggested that miR-362-5p might be a therapy target of GDM.

## Graphical abstract

**Figure:**
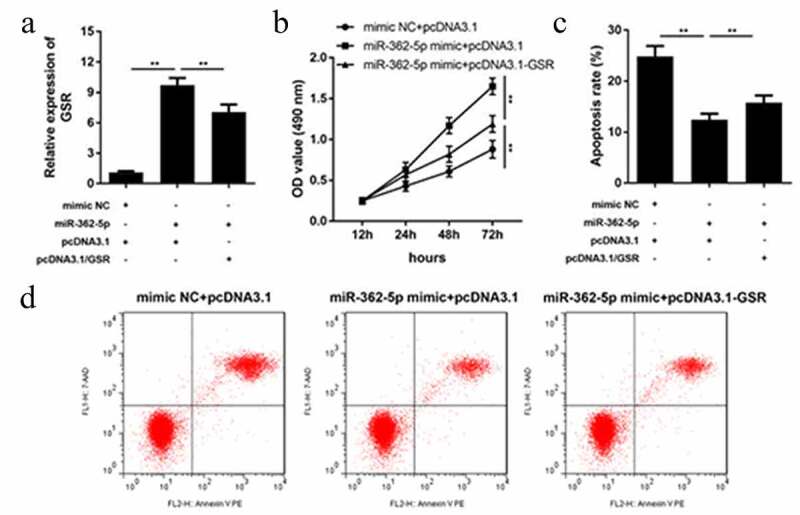

## Introduction

Gestational diabetes mellitus (GDM), as a kind of complications of pregnancy that is induced by insulin resistance and dysfunction of pancreatic β-cell during pregnancy, is one major obstacle to affect fetal growth and maternal health [1]. GDM is associated with the increased risk of cardiovascular diseases and type 2 diabetes both in the women and child [2]. The pathogenesis of GDM is closely related to obesity, environmental and genetic factors [3]. Early discovery and treatment of GDM help to improve the health of women [4]. Recently, the standard screening method for GDM for pregnant women is 75 g oral glucose tolerance test (OGTT) after 24 weeks of gestation [5]. However, this method has disadvantages, such as fasting and poor repeatability. Therefore, to explore an effective strategy for prevention, early screening, diagnosis and treatment of GDM is of vital essence.

MicroRNAs (miRNAs) are small, endogenous molecules with 19–25 nucleotides in size. MiRNAs regulate gene expression via binding to the 3ʹ untranslated region (3ʹUTR) of its targets [6]. Currently, more than 600 miRNAs have been identified in human placenta, and their dysregulation is involved in the physiological and pathological processes of placenta [7]. In patients with GDM, abnormal expression of miRNAs can be found in placenta, which are associated with GDM development [8]. MiR-362-5p is firstly discovered in the testes of primates [9]. Moreover, abnormal expression of miR-362-5p participates in the development of numerous diseases, including malignant tumors, diabetes mellitus, as well as essential hypertension [10–12]. Li et al. has unveiled that miR-362-5p expression is downregulated in GDM placenta tissue samples [13]. However, the underlying mechanisms have not been revealed.

In the present study, we aimed to investigate the functions of miR-362-5p and the potential molecular mechanism. High glucose (HG) inhibited the cellular function of trophoblast cells. Furthermore, miR-362-5p modulated the proliferation and apoptosis of HG-treated HTR-8/SVneo cells via targeting glutathione reductase (GSR). These findings showed that miR-362-5p might have the potential to treat GDM.

## Materials and methods

### Human placenta tissues

The protocol was authorized by the Ethics Committee of Chongqing Maternal and Child Health Hospital. Placentas were collected from women with GDM (n = 40) and healthy pregnant women (n = 40) from January 2019 to January 2020. Placenta villous tissue samples were collected after delivery immediately and stored at −80°C. Pregnant women at 24–28 weeks of gestation were tested for GDM by OGTT. The diagnostic criteria of GDM are as follows: fasting blood glucose >5.1 mmol/L, and/or 1-h glucose >10.0 mmol/L, and/or 2-h glucose >8.5 mmol/L. The exclusion criteria were as follows: pre-gestational diabetes mellitus, pre-gestational hypertension, chronic liver and kidney diseases, cancers, and other pregnancy complications. Each participant provided written informed consent before study. The clinical information is shown in Table 1.

**Table 1.** Clinical information of participator with or without GDM

Characteristics	Control (*n* = 40)	GDM (*n* = 40)	*P*-value
Age (year)	31.25 ± 2.25	31.72 ± 2.15	0.3424
BMI (kg/m^2^)	21.13 ± 2.98	27.44 ± 3.83	<0.0001
Gestational age (weeks)	39.82 ± 1.98	39.47 ± 2.02	0.6091
Fasting plasma glucose (mM)	4.38 ± 0.42	5.58 ± 0.52	<0.0001
1 h plasma glucose (mM)	6.65 ± 0.30	10.32 ± 0.48	<0.0001
2 h plasma glucose (mM)	5.63 ± 0.34	8.39 ± 0.26	<0.0001
Fetal birth weight (kg)	3.36 ± 0.32	3.42 ± 0.25	0.3529

### Cell culture and treatment

The human placenta trophoblast cells (HTR-8/SVneo) and HEK293T cells were both collected from ATCC (Manassas, VA, USA). All these cells were cultured at 37°C with 5% CO_2_ in RPMI-1640 containing glucose (1000 mg/l; Sigma-Aldrich, St. Louis, MO, USA), 10% FBS, and 1% double antibiotic (Procell life Science&Technology Co., Ltd, Wuhan, China).

D-glucose was obtained from Sigma-Aldrich. Cells were divided into two groups: high glucose (HG) and the control. The glucose concentration reached 25 mmol/l as HG group, and the glucose concentration in control group is 5 mmol/l [14].

### Dual-luciferase reporter assay

We used TargetScan Human 7.2 to predict the potential targets of miR-362-5p. Wild-type (wt)-GSR 3ʹUTR mRNA fragment and mutant (mut)-GSR 3ʹUTR were cloned into pMIR-Reporter Luciferase vectors (Ambion, Austin, TX, USA) individually. MiR-362-5p mimic and mimic NC were purchased from Shanghai GenePharma Co., Ltd (China). For luciferase reporter assay, HEK293T cells (5 × 10^5^ cells/well), cultured in complete medium, seeded into 24-well plates. After 24 h, HEK293T cells were transfection of mimic plasmids together with wt-GSR or mut-GSR using Lipofectamine 2000 (Invitrogen, Carlsbad, CA, USA). 24 h later, a Dual-Lucy Assay Kit (Solarbio, Beijing, China) was performed to detect the relative luciferase activity.

### Biotin-miRNA pull-down assay

Biotin labeled miR-362-5p (biotin-miR-362-5p) and nc (biotin-nc) were transfected into cells and harvested 72 h later. After treating with lysis buffer (Ambion), the lysates were incubated with Hydrophilic Streptavidin Magnetic Beads (genecompany, Shanghai, China) at 4°C for 3 h. Then the mixture was washed with washing buffer (lysis buffer, low salt buffer and high salt buffer). The enrichment of GSR was detected using qPCR.

### Cell transfection

MiR-362-5p mimic and mimic NC were acquired from Shanghai GenePharma Co., Ltd (China). pcDNA3.1-GSR and pcDNA3.1 were acquired from Invitrogen (USA). Transient transfection of HG-treated cells was conducted by Lipofectamine 2000 (Invitrogen). 24 h later, the cells were harvested to perform subsequent experiments.

### Cell proliferation assay

Cell viability was determined by a Cell Counting Kit-8 (WST-8/CCK-8; Abcam, Cambridge, MA, USA). Cells were seeded into 96-well plates at the density of 1 × 10^5^ cells/well and cultured at specific time (12, 24, 48 and 72 h). 10 µl CCK-8 was added to the cells and cultured for 3 h. The absorbance value was measured at 490 nm using a microscope (Bio-TEK, Winooski, VT, USA).

### Flow cytometry

Cell apoptosis was analyzed by Annexin V-PE Cell Apoptosis Detection Kit (Beyotime Biotechnology, Shanghai, China). Following washing with PBS, transfected cells at the density of 5 × 10^4^ cells/ml were re-suspended using Annexin V-PE (195 µl). Afterward, 7-AAD (5 µl) was added and incubated for 20 min in dark. Subsequently, cell apoptosis was detected using a flow cytometry.

### qPCR

To detect miRNA expression, total RNA in patients’ tissues and HTR-8/SVneo cells was extracted with miRNeasy FFPE Kit (QIAGEN, Hilden, Germany) and reversed transcription was carried out by miScript II RT kit (Qiagen). To analyze mRNA expression, total RNA was extracted using RNeasy Mini Kit (Qiagen), and cDNA first chain was synthesized by PrimeScriptTM RT reagent Kit (Takara, Dalian, China). qPCR was carried out using Power SYBR Green PCR Master Mix (Applied Biosystems, Foster City, CA, USA). qPCR was performed on ABI 7500 Real-Time PCR System (Applied Biosystems) with the following cycle conditions: 95 °C for 10 min (pre-denaturation), 40 cycles of 95°C for 15 sec (denaturation) and 60°C for 1 min (annealing/extend). U6 and GAPDH were the normalization of miR-362-5p and mRNA, respectively. Relative expression was determined by 2^−ΔΔCt^ method. The sequences of specific primer were: miR-362-5p at mature stage (F: 5ʹ-GTCACGAAATCCTTGGAACCTAG-3ʹ, R: 5ʹ-TATGGTTGTTCTCGTCTCCTTCTC-3ʹ), U6 snRNA (F: 5ʹ-CTCGCTTCGGCAGCACA-3ʹ, R: 5ʹ-AACGCTTCACGAATTTGCGT-3ʹ.), GSR (F: 5ʹ-TCGGAATTCATGCACGATCA-3ʹ, R: 5ʹ-GGCTCACATAGGCATCCCTTT-3ʹ.), and GAPDH (F: 5ʹ-GAAGGTGAAGGTCGGAGTCA-3ʹ, R: 5ʹ- TTCACACCCATGACGAACAT-3ʹ).

### Western blot

The protein was extracted from HTR-8/SVneo cell line using RIPA Lysis Buffer (Thermo Fisher Scientific) on ice after washing with PBS. Pierce BCA Protein Assay Kit (Thermo Fisher Scientific) was conducted to test the concentration of protein. The protein (30 μg) was separating by 10% SDS-PAGE, transferred to PVDF membranes, and followed by blocking using 5% not-fat milk. The membranes were incubated with primary antibodies at 4°C overnight. After washing with PBS, the membranes were incubated with secondary antibody at room temperature for 1 h. All bands were visualized by Beyoecl Plus ECL Kit (Beyotime Biotechnology). Relative expression was calculated by protein/GAPDH ratio based on band intensity using Image J software.

Primary and secondary antibodies used here including anti-PI3K (ab191606, 1:1,000), anti-AKT (ab179463, 1:10,000), anti-p-PI3K (ab182651, 1:1,000), anti-p-AKT (ab38449, 1:1,000), anti-bax (ab182733, 1:2000), anti-caspase3 (ab13847, 1:500), anti-cleaved caspase3 (c-caspase3; ab2302, 1:500), anti-bcl-2 (ab182858, 1:2,000), anti-GAPDH (1:2,500) and goat anti rabbit IgG H&L (HRP) (ab205718, 1:10,000). All antibodies were obtained from Abcam, USA.

### Statistical analysis

All test data were assessed by GraphPad Prism 6.0 (La Jolla, CA, USA), and presented as mean ± SD. Differences in groups were compared using student’s t-test and one-way ANOVA. Pearson Correlation coefficient was utilized to analyze miR-362-5p and GSR relationship. P < 0.05 was considered significantly different.

## Results

Herein, to investigate the roles of miR-362-5p in GDM, we revealed the effects of miR-362-5p on cellular processes of trophoblast cells. We found that miR-362-5p was downregulated and its target GSR was upregulated in GDM patients. Furthermore, miR-362-5p/GSR axis promoted the proliferation and suppressed apoptosis of HG-treated HTR-8/SVneo cells.

### HG exposure induces HTR-8/SVneo cell dysfunction and miR-362-5p downregulation

**Figure 1.**
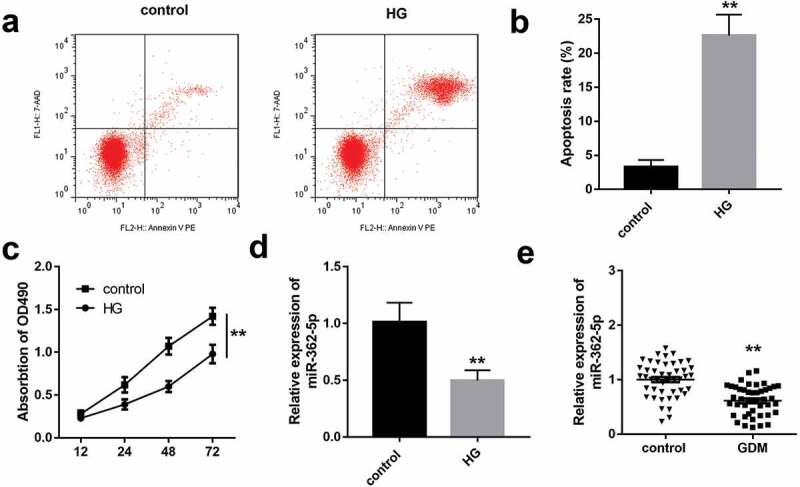
The impacts of HG treatment on proliferation, apoptosis, and miR-362-5p level in HTR-8/SVneo cells. (a) Cell apoptosis was detected by flow cytometry after treating with HG. (b) Quantification of A. (c) The proliferation of HG-treated cells was determined by CCK-8. (d) MiR-362-5p expression was tested by qPCR in HTR-8/SVneo cells treated with or without HG. (e) The level of miR-362-5p was tested in placenta tissues from healthy (*n* = 40) or GDM women (*n* = 40) through qPCR. **P < 0.01

HTR-8/SVneo cells exposed to 25 mM glucose as the HG group, and 5 mM glucose as the control group. As illustrated in Figure 1a-c, HG significantly inhibited the proliferation and induced apoptosis of HTR-8/SVneo cells. Moreover, miR-362-5p level was significantly decreased in HG-treated HTR-8/SVneo cells compared with control group (Figure 1d). Similarly, the data of qPCR demonstrated that miR-362-5p expression was significantly downregulated in GDM placenta tissues (Figure 1e).

### Overexpressed miR-362-5p enhances the proliferation and represses cell apoptosis

**Figure 2.**
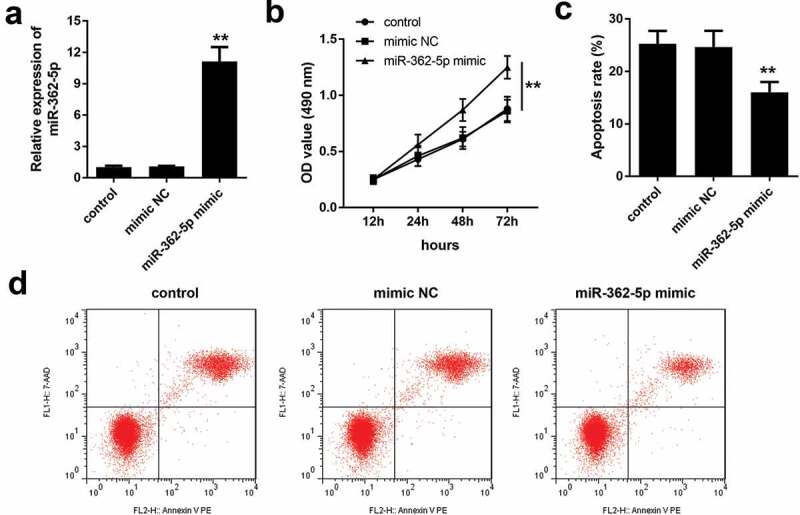
MiR-362-5p overexpression enhanced the proliferation and inhibited apoptosis. (a) Transfection efficiency was measured by qPCR post-transfection. (b) CCK-8 was used to evaluate cell proliferation and (c–d) flow cytometry was carried out to evaluate cell apoptosis in transfected cells. **P < 0.01

After transfection of miR-362-5p mimic, the expression of miR-362-5p was significantly increased, compared with mimic NC group (Figure 2a). The results of CCK-8 assay and flow cytometry indicated that compared with mimic NC group, miR-362-5p overexpression significantly facilitated the proliferation but inhibited apoptosis of HG-treated HTR-8/SVneo cells (Figure 2b-d).

### MiR-362-5p targets to GSR

**Figure 3.**
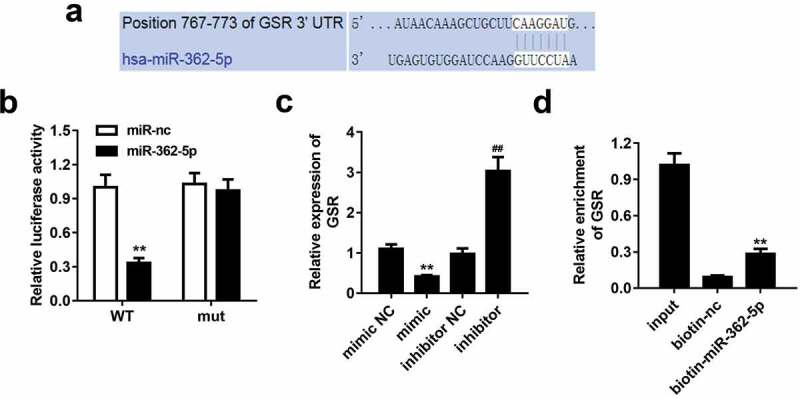
MiR-362-5p could target to GSR. (a) The binding sites of miR-362-5p on 3ʹUTR region of GSR was predicted. (b) Relative luciferase activity was measured when cells co-transfected with mimic plasmids together with wt GSR 3ʹUTR and mut 3ʹUTR of GSR. (c) GSR expression was detected by qPCR after transfection. (d) The enrichment of GSR was measured using Biotin-miRNA pull-down assay. **P < 0.01, ##*P* < 0.01

According to data predicted by bioinformatics tool TargetScan, miR-362-5p could bound to 3ʹUTR region of GSR (Figure 3a). The data of dual-luciferase reporter assay showed that wt-GSR 3ʹUTR and miR-362-5p mimic co-transfected significantly decreased luciferase activity compared with miR-NC mimic group. However, wt-GSR 3ʹUTR or mut-GSR 3ʹUTR did not influence luciferase activity while co-transfected with miR-NC mimic (Figure 3b). Overexpressed miR-362-5p induced the downregulation of GSR, which was upregulated by inhibition of miR-362-5p, compared with corresponding NC groups, respectively (Figure 3c). Additionally, GSR level was enriched in biotin-miR-362-5p group (Figure 3d).

### GSR expression is elevated in GDM placenta tissues

**Figure 4.**
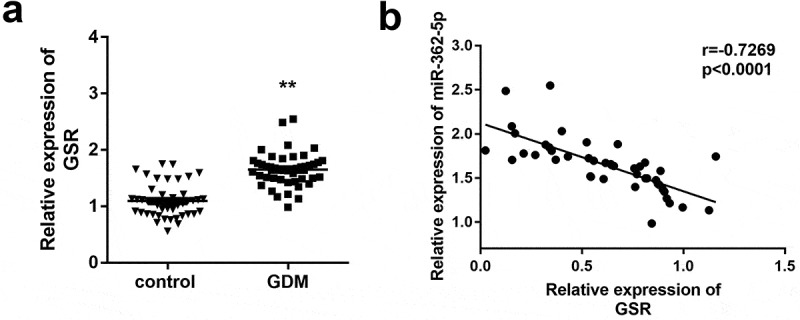
Upregulation of GSR expression was measured in placenta tissues of GDM. (a) GSR expression was tested in placenta tissues from 40 healthy and 40 GDM women by qPCR. (b) MiR-362-5p and GSR relationship in GDM placenta tissues was determined by Pearson correlation coefficient. **P < 0.01

GSR level was further measured in tissue samples, and the data demonstrated GSR level was significantly increased in GDM patients compared with healthy control (Figure 4a). Moreover, according to the data of Pearson correlation coefficient, the expression of miR-362-5p was negatively correlated with GSR in placenta tissues from patients with GDM (r = −0.7269, p < 0.0001; Figure 4b).

MiR-362-5p regulates cell proliferation and apoptosis via targeting GSR

**Figure 5.**
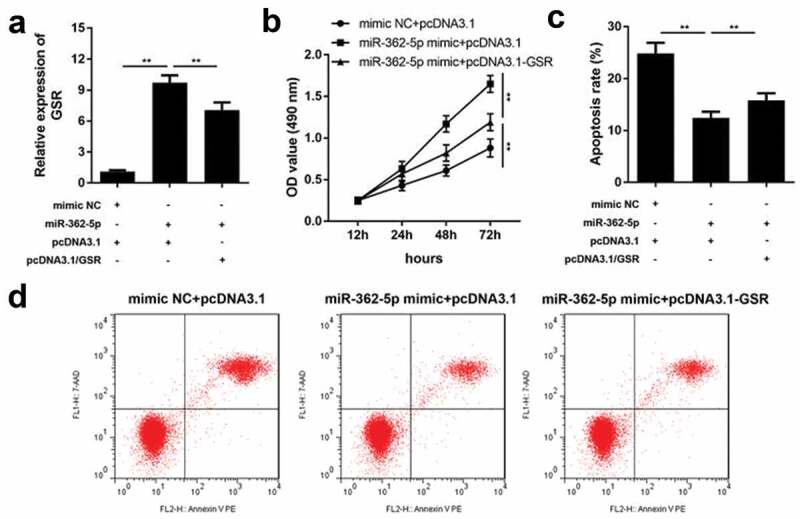
Enforced level of GSR rescued miR-362-5p induced effects on cell proliferation and apoptosis. (a) Transfection efficiency was measured by qPCR after transfection. (b) Cell proliferation was analyzed using CCK-8 in cells transfection of miR-362-5p mimic and pcDNA3.1-GSR. (c–d) Flow cytometry was performed to assess cell apoptosis after miR-362-5p mimic and pcDNA3.1-GSR transfected into HG-treated HTR-8/SVneo cells. Apoptosis rate was analyzed. **P < 0.01

After transfection for 24 h, transfection efficiency was determined by qPCR. The results demonstrated that GSR level was significantly upregulated by miR-362-5p, which was abolished by pcDNA3.1-GSR (Figure 5a). MiR-362-5p-induced proliferation was suppressed by GSR overexpression (Figure 5b). Moreover, overexpressed GSR significantly abolished the suppression of apoptosis induced by miR-362-5p in cells (Figure 5c and d).

### MiR-362-5p targets GSR to influence PI3K/AKT pathway and apoptosis-related factors

**Figure 6.**
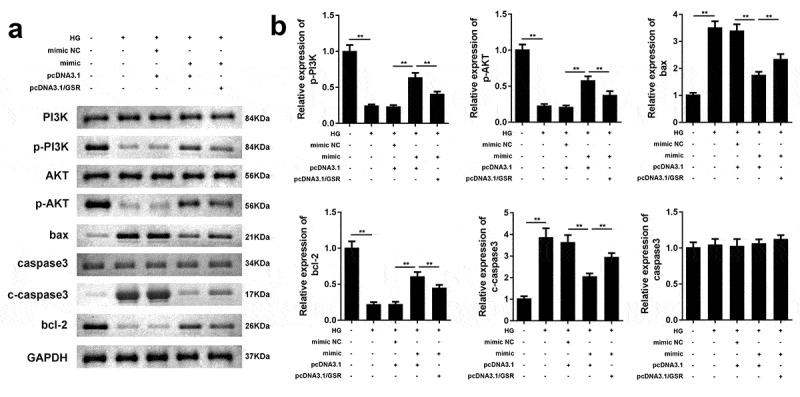
MiR-362-5p affected PI3K/AKT signaling and bax, cleaved caspase3, and bcl-2 expression via targeting GSR. (a) The protein expression of each signaling was determined by western blot. (b) Quantification of the relative protein expression normalized to GAPDH. **P < 0.01

As illustrated in Figure 6a and b, HG treatment decreased phosphorylation of PI3K and AKT, meanwhile enhanced bax, cleaved caspase3 and reduced bcl-2, which was reversed by overexpressed miR-362-5p. However, the regulatory roles of miR-362-5p on the expression of p-PI3K, p-AKT, bax, cleaved caspase3, and bcl-2 were antagonized by GSR. Total levels of PI3K, AKT and caspase3 were not affected by HG treatment, miR-362-5p or GSR.

## Discussion

Placenta is an organ for nutrient exchange and metabolism between mother and fetus. In GDM, a large amount of blood glucose enters into the fetal circulation through the placenta [15]. HG could induce lipid metabolism in human placenta [16]; regulate endothelial dysfunction [17]; reduce fatty acid and increase triglyceride [18]. In the present study, HG inhibited the proliferation and induced apoptosis of placental trophoblast cells, suggesting that HG is associated with GDM.

MiRNAs are considered to be associated with the progression of human diseases [8,19]. Various miRNAs are involved in GDM. For example, miR-657 promotes proliferation, migration, and polarization through targeting FAM46C in placenta macrophages [20]. Additionally, miR-142-3p overexpressed enhances the proliferation and suppresses apoptosis of pancreatic β cells [21]. MiR-362-5p plays important roles in a large number of human diseases. For example, in acute myeloid leukemia, miR-362-5p is a prognostic biomarker, in which high expression is associated with poor survival rate, and promotes proliferation and cell cycle progress [10,22]. However, the roles of miR-362-5p vary with cancer and cell types. MiR-362-5p was highly expressed in NSCLC, which accelerates the migration, invasion, colony formation and tumor growth [23]. Inhibition miR-362-5p expression suppresses cell proliferation, migration, invasion, but facilitates apoptosis of breast cancer [24]. Inversely, miR-362-5p was identified as an anti-tumor miRNA in renal cell carcinoma and neuroblastoma [25,26]. In GDM, a previous study has reported that miR-362-5p level in placenta tissues was reduced, and participated in regulating EGFR/PI3K/AKT signaling pathway [7]. In the current study, miR-362-5p level was decreased in GDM placenta tissues, which is consistent with previous study [13]. Moreover, overexpressed miR-362-5p accelerated the proliferation and inhibited the apoptosis of HG-treated HTR-8/SVneo cells, suggesting that miR-362-5p may restore the cellular functions of placental trophoblast cells.

GSR maintains cellular redox hmoeostasis. It not only maintains reductive glutathione supply, but also regulates reactive oxygen [27]. In addition, GSR is involved the compensating antioxidant pathways dependent on thiol, which maintain the balance between protein dithiol and disulfide [28]. Except that, GSR protects the host against bacterial pathogen by promoting neutrophil bactericidal activity [29]. GSR gene in human is located in chromosome 8p12, which is usually lost in cancers [28,30,31]. However, the functions of GSR on the proliferation and apoptosis are little known. A previous study has reported that GSR level is upregulated in placenta of GDM [32]. In the current study, miR-362-5p could target to GSR, which expression was upregulated in GDM placenta tissues and negatively correlated with miR-362-5p both in tissues and cells. Moreover, enforced GSR level rescued the promotion of proliferation and suppression of apoptosis induced by miR-365p in HG-treated HTR-8/SVneo cells. These findings mentioned above suggested that miR-362-5p accelerated proliferation and inhibited apoptosis via targeting GSR.

PI3K/AKT signaling pathway contributes to biological processes such as proliferation, apoptosis, and metabolism [33]. In GDM, miR-351 prevents insulin resistance and liver gluconeogenesis via PI3K/AKT pathway [34]. Additionally, miR-29b reduces blood glucose through targeting PI3K/AKT signaling [35]. Bcl-2 is an anti-apoptosis factor, and bax and cleaved caspase3 are pro-apoptosis factors. In GDM, miR-142-3p inhibited apoptosis through increasing bcl-2 expression and reducing bax and cleaved caspase3 [21]. In our study, miR-362-5p enhanced p-PI3K, p-AKT, bcl-2 levels, and suppressed bax and cleaved caspase3. However, GSR abolished the effects induced by miR-362-5p. These findings suggested that miR-362-5p effected on PI3K/AKT pathway, bcl-2, bax, and cleaved caspase3 through targeting GSR.

## Conclusion

HG restrained the proliferation and facilitated apoptosis. MiR-362-5p level is downregulation in GDM placental tissues and HG-treated cells. GSR is identified as a direct target of miR-362-5p. Overexpressed miR-362-5p directly targeted GSR to promote proliferation and suppress apoptosis of HTR-8/SVneo cells via activating PI3K/AKT signaling pathway, enhancing bcl-2 and repressing bax and cleaved caspase3. The study provided a theoretical foundation for the treatment of GDM.

## Supplementary Material

Supplemental Material

## Research highlights

1. MiR-362-5p level was upregulated in placenta tissues of patients with GDM.

2. High glucose inhibited HTR-8/SVneo cell proliferation and promoted apoptosis.

3. MiR-362-5p accelerated the proliferation and repressed apoptosis of HG-treated cells.

4. GSR was found as a target of miR-362-5p.

5. MiR-362-5p played biological functional roles through targeting GSR.
