# Supplementary material for: MicroRNA-362-5p promotes the proliferation and inhibits apoptosis of trophoblast cells via targeting glutathione-disulfide reductase
Source: Bioengineered. 2021 Jun 9;12(1):2410–9. doi: 10.1080/21655979.2021.1933678 (PMC8806602; doi:10.1080/21655979.2021.1933678)
Supplement: Supplemental Material [file KBIE_A_1933678_SM6564.zip › Supplementary/downloadFromZipFile.pdf]

The full code and data can be accessed under:
https://box.uke.de/s/cCD3Oyopqkl7GNv
Password: dimeimmune

DIMEimmune: Robust estimation of infiltrating lymphocytes in CNS tumors from DNA methylation profiles

Sepehr Safaei, Michael Bockmayr

23 Okt 2020, 19:51

Loading Required Libraries

```
library(minfi)
```

```
## Loading required package: BiocGenerics
```

```
## Loading required package: parallel
```

```
##
## Attaching package: 'BiocGenerics'
```

```
## The following objects are masked from 'package:parallel':
##
##     clusterApply, clusterApplyLB, clusterCall, clusterEvalQ,
##     clusterExport, clusterMap, parApply, parCapply, parLapply,
##     parLapplyLB, parRapply, parSapply, parSapplyLB
```

```
## The following objects are masked from 'package:stats':
##
##     IQR, mad, sd, var, xtabs
```

```
## The following objects are masked from 'package:base':
##
##     anyDuplicated, append, as.data.frame, basename, cbind,
##     colnames, dirname, do.call, duplicated, eval, evalq, Filter,
##     Find, get, grep, grepl, intersect, is.unsorted, lapply, Map,
##     mapply, match, mget, order, paste, pmax, pmax.int, pmin,
##     pmin.int, Position, rank, rbind, Reduce, rownames, sapply,
##     setdiff, sort, table, tapply, union, unique, unsplit, which,
##     which.max, which.min
```

```
## Loading required package: GenomicRanges
```

```
## Loading required package: stats4
```

```
## Loading required package: S4Vectors
```

```
##
## Attaching package: 'S4Vectors'
```

```
## The following object is masked from 'package:base':
##
##     expand.grid
```

```
## Loading required package: IRanges
```

```
## Loading required package: GenomeInfoDb
```

```
## Loading required package: SummarizedExperiment
```

```
## Loading required package: Biobase
```

```
## Welcome to Bioconductor
##
##     Vignettes contain introductory material; view with
##     'browseVignettes()'. To cite Bioconductor, see
##     'citation("Biobase")', and for packages 'citation("pkgname")'.
```

```
## Loading required package: DelayedArray
```

```
## Loading required package: matrixStats
```

```
##
## Attaching package: 'matrixStats'
```

```
## The following objects are masked from 'package:Biobase':
##
##     anyMissing, rowMedians
```

```
## Loading required package: BiocParallel
```

```
##
## Attaching package: 'DelayedArray'
```

```
## The following objects are masked from 'package:matrixStats':
##
##     colMaxs, colMins, colRanges, rowMaxs, rowMins, rowRanges
```

```
## The following objects are masked from 'package:base':
##
##     aperm, apply, rowsum
```

```
## Loading required package: Biostrings
```

```
## Loading required package: XVector
```

```
##
## Attaching package: 'Biostrings'
```

```
## The following object is masked from 'package:DelayedArray':
##
##     type
```

```
## The following object is masked from 'package:base':
##
##     strsplit
```

```
## Loading required package: bumphunter
```

```
## Loading required package: foreach
```

```
## Loading required package: iterators
```

```
## Loading required package: locfit
```

```
## locfit 1.5-9.1    2013-03-22
```

```
## Registered S3 method overwritten by 'openssl':
##   method      from
##   print.bytes Rcpp
```

```
## Setting options('download.file.method.GEOquery'='auto')
```

```
## Setting options('GEOquery.inmemory.gpl'=FALSE)
```

Functions

```
idatPaths <- function(idatFolderPath){
  idatFilesList <- list.files(idatFolderPath)
  idatNames <- gsub("_Grn.*$","",idatFilesList[grep("Grn", idatFilesList)])
  file.path(idatFolderPath, idatNames)
}

methValNoob <- function(idatFolderPath){
  idatPaths <- idatPaths(idatFolderPath)
  rgset <- read.metharray(idatPaths, force = TRUE)
  prgset <- preprocessNoob(rgset)
  getBeta(prgset)
}
```

Loading the Signatures and the Rotation Matrices

```
# set the folder Supplement as work directory
setwd("/mnt/scratch1/sepehr/Supplement")

# loading
load("DIMEimmune.RData")

# The definition of the loaded parameters:

# DIME_CD4: signature of CD4+ T cells
# DIME_CD8: signature of CD8+ T cells
# DIME_TIL: signature of Tumor Infiltrating Lymphocytes
# PCA_CD4: rotation matrix and standard deviation of principal component for DIME_CD4
# PCA_CD8: rotation matrix and standard deviation of principal component for DIME_CD8
# PCA_TIL: rotation matrix and standard deviation of principal component for DIME_TIL
```

DIMEimmune Algorithm

```
meth <- methValNoob("Samples")
```

```
## Loading required package: IlluminaHumanMethylation450kmanifest
```

```
## Loading required package: IlluminaHumanMethylation450kanno.ilmn12.hg19
```

```
CD4 <- -predict(PCA_CD4,t(meth[DIME_CD4,]))[,1]
CD8 <- -predict(PCA_CD8,t(meth[DIME_CD8,]))[,1]
TIL <- predict(PCA_TIL,t(meth[DIME_TIL,]))[,1]
```
