# Supplementary figures and images for: MicroRNA-362-5p promotes the proliferation and inhibits apoptosis of trophoblast cells via targeting glutathione-disulfide reductase
Source: Bioengineered. 2021 Jun 9;12(1):2410–9. doi: 10.1080/21655979.2021.1933678 (PMC8806602; doi:10.1080/21655979.2021.1933678)

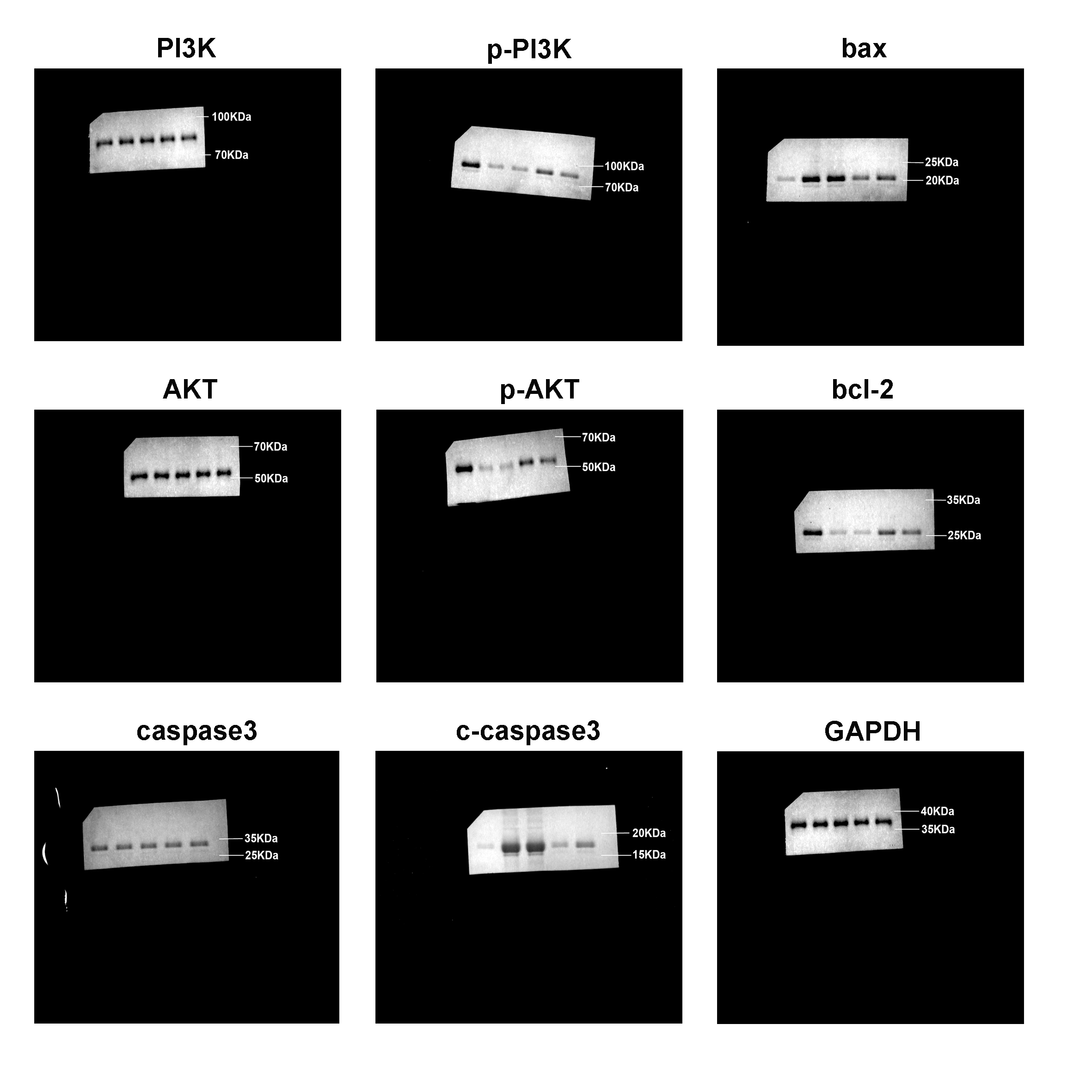

Supplement: Supplemental Material [file KBIE_A_1933678_SM6564.zip › Supplementary/supplementary materials.jpg]
